# Case report: Metastatic pancreatic cancer to bilateral testes and spermatic cords

**DOI:** 10.1016/j.eucr.2026.103419

**Published:** 2026-03-23

**Authors:** Matthew Phillipi, Sidney Roberts, David Yao

**Affiliations:** a David Geffen School of Medicine, University of California at Los Angeles, USA; b Univeristy of Texas Southwestern Medical Center, USA

**Keywords:** Metastatic pancreatic cancer, Testicular cancer, Spermatic cord cancer

## Abstract

We present a 65-year-old man with pancreatic cancer treated with chemotherapy and chemoradiation for local control, who presented three years later with groin pain and a retracted right testis. Evaluation revealed pancreatic ductal adenocarcinoma metastatic to bilateral testes and spermatic cords with rising CA19-9. He underwent palliative bilateral radical orchiectomy. We present this case of metastatic pancreatic cancer to bilateral testes and spermatic cords as the first report in the English literature. This case highlights the need to consider metastatic disease in men over 40 presenting with a new testicular or spermatic cord mass and a history of malignancy.

## 1 Introduction

Approximately one third of testicular masses are malignant, of which 6–8% are metastatic deposits. Metastatic tumors to the testis are uncommon, possibly due to the protection offered by the blood–testis barrier. The most common primary sources of metastatic solid tumors to the testis are prostate, lung, and melanoma.^1^ Metastasis of pancreatic adenocarcinoma to the testis is an extremely rare finding with fewer than 20 reported cases in the English literature.^2^ Malignant tumors of the spermatic cords are also uncommon, representing 25% of all spermatic cord tumors. The majority of spermatic cord tumors are benign lipomas.^3^ To our knowledge, this is the first reported case in the English literature of metastatic pancreatic cancer involving bilateral testes and spermatic cords.

## 2 Case presentation

The patient is a 65-year old man with pancreatic adenocarcinoma. CT scan revealed a 3.1 × 2.5 cm mass in the head of the pancreas involving the portal vein and the superior mesenteric artery. The tumor was deemed unresectable and he was started on combination chemotherapy with FOLFIRINOX until treatment was stopped for cytopenia and neuropathy. He received 40Gy radiation to the pancreas and the chemotherapy regimen was switched to Xeloda. He remained on Xeloda for approximately 2 years until he presented with rising CA 19-9 (36 to 118 U/mL in 2 months) along with groin pain.

**Fig. 1:**
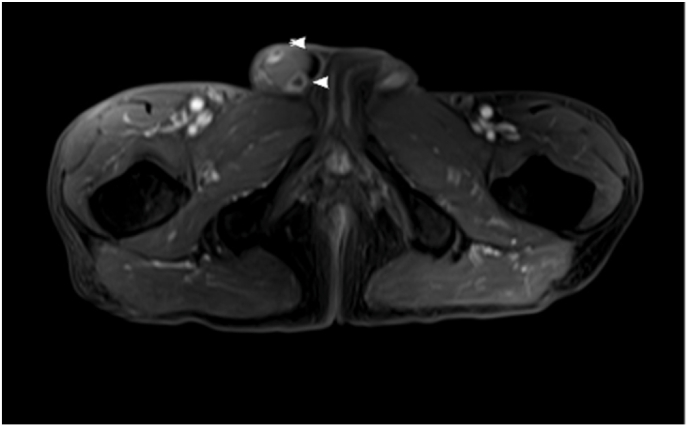
MRI identified right inguinal testis with adjacent rim enhancing nodules (arrowheads).

Three years later, the patient presented to Urology. He was found to have a retracted right testicle upon examination. Testicular ultrasound and pelvic MRI showed enhancing masses in the right inguinal canal [Fig. 1].

**Fig. 2:**
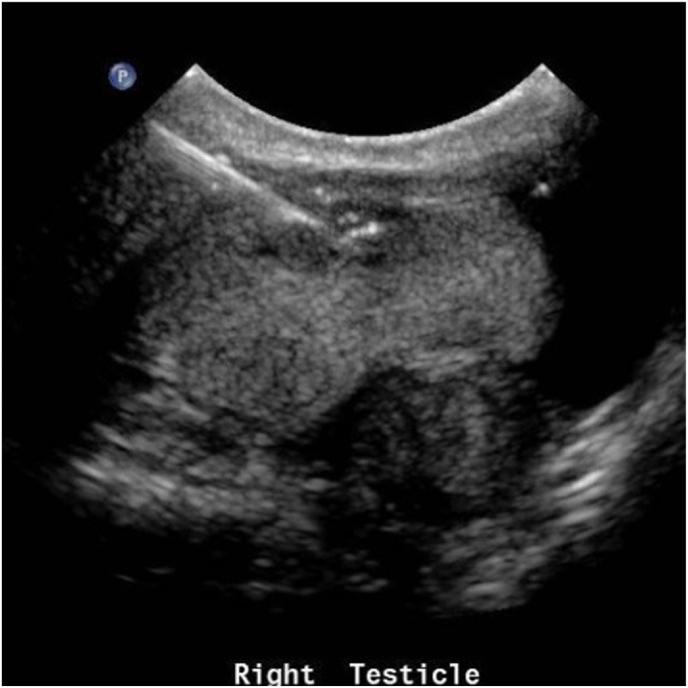
US guided biopsy with needle within solid testicular mass.

Due to the concern for metastatic disease, he had an ultrasound guided biopsy which confirmed the presence of malignant cells favoring adenocarcinoma [Fig. 2].

The patient was scheduled for palliative right radical orchiectomy. Prior to surgery, the patient expressed a clear preference for maximal debulking of his scrotal disease for palliative purposes. On preoperative examination, the right testis was adherent to the superior scrotal wall, and an associated extratesticular mass was identified within the right hemiscrotum. These findings raised strong concern for local invasion of tumor into the scrotal wall. Following a detailed discussion and shared decision-making with the patient, the surgical plan included right radical orchiectomy, right hemiscrotectomy, and right inguinal lymphadenectomy.

Hemiscrotectomy was performed due to intraoperative evidence of tumor adherence and matting between the testis and the overlying scrotal tissue, raising concern for direct local extension. En bloc resection of the involved hemiscrotal tissue was therefore performed to achieve maximal local disease control.

During the procedure, a firm, non-mobile mass was palpated along the left spermatic cord. This finding had not been present on physical examination or cross-sectional imaging performed approximately one month prior to surgery. Given the patient's previously expressed wishes for aggressive palliative debulking, a discussion was held intraoperatively with the patient's family. After reviewing the findings and goals of care, the decision was made to proceed with left radical orchiectomy in order to achieve the patient's stated objective of maximal disease debulking.

Post-operatively, the patient confirmed that the surgical management aligned with his wishes and reported significant improvement in his groin and scrotal pain.

**Fig. 3:**
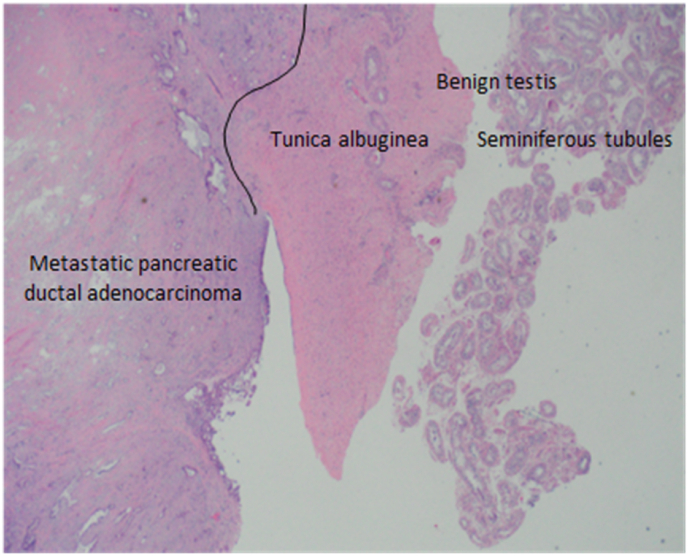
Metastatic pancreatic ductal adenocarcinoma involving testis.

**Fig. 4:**
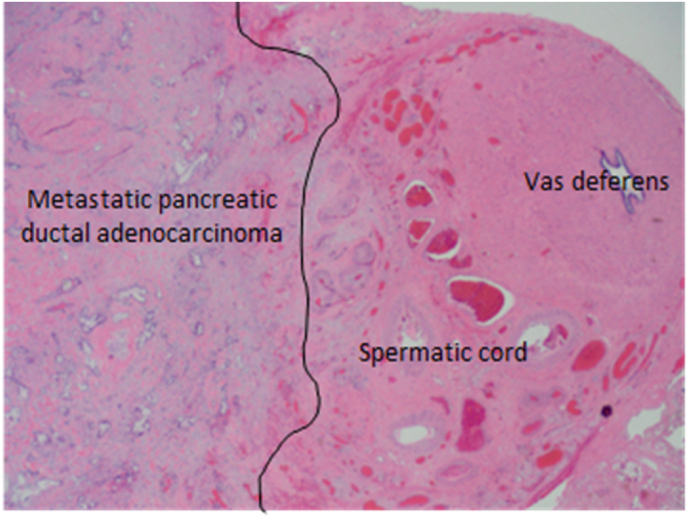
Metastatic pancreatic ductal adenocarcinoma involving spermatic cord.

Pathology revealed metastatic pancreatic ductal adenocarcinoma involving bilateral testes [Fig. 3] and spermatic cords [Fig. 4]. The right inguinal lymph nodes were negative for carcinoma. The tumor marker CA 19-9 did not improve after surgery (119 before surgery, 178 U/mL after surgery).

The patient was started post-operatively on lifelong testosterone replacement therapy and his chemotherapy was changed to gemcitabine and abraxane. This resulted in a significant decrease in his tumor marker CA 19-9 (102 U/mL) and improvement in quality of life with no evidence of metastatic deposits or disease progression 6 months after the orchiectomy.

## 3 Discussion

Metastatic cancer to the testis represents only 6–8% of malignant testicular masses. However, the percentage of testicular masses that are metastatic deposits peak in men after the 6th decade.^4^ The pancreas is a rare primary site of metastatic disease to the testis with less than 20 reported cases.

A review of the literature demonstrates most reported cases of pancreatic cancer to the testis have been unilateral masses to the right testicle, with only one other reported case involving both testes in the English literature. Previous reports are largely confined to the testis, with rare cases exhibiting involvement of the spermatic cord or epididymis.^3^ Very few cases have described metastasis to both the spermatic cord and testis, and these have been limited to unilateral presentations.^5^ To our knowledge, this case is unique as it is the first report of pancreatic adenocarcinoma metastatic to the bilateral testis and spermatic cords.

Pancreatic cancer metastasis to the testis may occur through hematogenous and lymphatic routes, with the proposed mechanism involving retrograde spread via venous and lymphatic obstruction caused by regional tumor extension. Pancreatic cancer cells possess epithelial-to-mesenchymal transition properties that facilitate escape into circulation and metastatic colonization.^6^ Regional spread of pancreatic carcinoma may create venous and lymphatic obstruction, leading to inferior passage of tumor emboli to the testis through retrograde flow along both venous and lymphatic routes.^7^ Tumor cells can also reach the testis via arterial routes, becoming entrapped in testicular capillaries where they may proliferate. While the reason for a right sided bias may be the result of chance, one hypothesis is that the later descent of the right testis may predispose it to spread via the remains of the tunica vaginalis testis.^4^

Metastasis of primary pancreatic cancer to the spermatic cords is an even rarer finding. The most common primary tumors metastasizing to the spermatic cords are neoplasms of the prostate and stomach.^3^ Similar to the mechanism described above, metastasis to the spermatic cords is thought to occur via retrograde lymphatic or hematic routes.^8^ The prognosis for metastatic pancreatic cancer is poor, with less than 1% of patients with metastatic pancreatic cancer surviving 5 years following diagnosis.^9^

In patients with testicular masses where primary testicular cancer is unlikely due to age (most common age of primary testicular cancer is 20–39),^10^ metastasis should be considered, especially if there is a history of or risk factors for prostate cancer, lung cancer, or melanoma. In a study involving 57 adult patients (age 18–85 years) who had metastatic disease to the testis, the median age of initial presentation was 57 years.^11^

While most tumors of the spermatic cord are benign, 25% are malignant neoplasms. Of the malignant tumors, only 8% are metastatic deposits.^12^ The mean age of incidence of metastatic spermatic cord tumors was 61 years in a study of 16 patients identified from 2000 to 2015.^3^ The prognosis of metastasis to the spermatic cords is poor. The 2 year survival was 36% in those 16 patients from 2000 to 2015. Palliative radical orchiectomy is the preferred treatment option for metastatic spermatic cord tumors even in patients who are being treated for their primary disease with chemotherapy.

While there have been a few reported cases of metastatic pancreatic cancer to the testis and spermatic cord, this is the first reported case in the English literature of pancreatic cancer involving bilateral testes and spermatic cords. This presentation shows that while metastasis to the testes and spermatic cords is rare, especially from a primary site such as the pancreas, it should be considered in men over 40 presenting with *de-novo* testicular masses with a history of neoplasm even if they have been previously treated for their primary disease.

## 4 Conclusion

While rare, this case demonstrates the possibility of metastatic pancreatic ductal adenocarcinoma to bilateral testes and spermatic cords. When evaluating older men presenting with *de-novo* testicular masses it is important to maintain suspicion for metastatic disease to ensure timely diagnoses and appropriate management.

## Informed consent statement

Informed consent was obtained from the patient involved in this case report.

## CRediT authorship contribution statement

**Matthew Phillipi:** Writing – review & editing, Writing – original draft, Project administration. **Sidney Roberts:** Writing – review & editing, Writing – original draft. **David Yao:** Writing – review & editing, Supervision.

## Funding source

This work was supported by the Winston Faculty Research Grant (grant number 45541).

## Declaration of competing interest statement

The authors declare they have no known competing financial interests or personal relationships that could have appeared to influence the work reported in this paper.
